# Evaluation of breastfeeding self-efficacy of puerperal women in shared rooming units

**DOI:** 10.1016/j.heliyon.2018.e00900

**Published:** 2018-11-02

**Authors:** Luciane Morelis de Abreu, Rosângela Filipini, Beatriz da Costa Aguiar Alves, Gláucia Luciano da Veiga, Fernando Luiz Affonso Fonseca

**Affiliations:** a Departamento de Enfermagem da Faculdade de Medicina do ABC, Av. Príncipe de Gales, 821, CEP 09060-650, Santo André, SP, Brazil; b Laboratório de Análises Clínicas da Faculdade de Medicina do ABC, Av. Príncipe de Gales, 821, CEP 09060-650, Santo André, SP, Brazil; c Departamento de Ciências Farmacêuticas da Universidade Federal de São Paulo, UNIFESP, Rua Prof. Artur Riedel, 275, CEP 09972-270, Diadema, São Paulo, Brazil

**Keywords:** Public health

## Abstract

**Purpose:**

To evaluate the perception of breastfeeding self-efficacy in puerperal women and to verify the association between Breastfeeding Self-Efficacy Scale-Brazilian Version (BSES-VB) scores and sociodemographic and obstetric variables. The practice of breastfeeding is of extreme importance, not only because of its affective value but also because the nutritional composition of human milk is essential nutrients for the adequate growth and development of the newborn.

**Design and methods:**

This is a quantitative and cross-sectional study. The sample consisted of 100 puerperal women. The research instruments used were form with demographic, economic, obstetric and breastfeeding data and the BSES-VB.

**Results:**

40% of the mothers obtained scores compatible with average breastfeeding self-efficacy, 35% of them presented high efficacy and 25% low efficacy. There was no significant relationship between sociodemographic and obstetric variables with the BSES-VB mean scores in the puerperal period. In the association between some variables and items of the breastfeeding self-efficacy scale, it was observed that breastfeeding guidance in the prenatal period and marital status were statistically significant with the mean scores of each item on the scale.

**Conclusion:**

Only 35% of the puerperal women presented high breastfeeding efficacy. Breastfeeding guidance variables in prenatal care and marital status were statistically significant.

**Practice implications:**

the work of the professionals who work in the care of puerperal women becomes of great importance to provide information on the breastfeeding theme and determining the adherence to the practice of breastfeeding.

## 1 Introduction

The practice of breastfeeding is of extreme importance, not only because of its affective value about the bond between mother and baby, but because the nutritional composition of human milk (HM) is balanced, consisting of essential nutrients which are important for the adequate growth and development of the newborn (Grando and Zuse, 2011). Due to its many benefits, it is recommended that babies up to the age of six months be fed exclusively on breast milk (BM), which should be supplemented with other foods until the age of two or more years (WHO, 2008). This is considered throughout the world to be paramount for child health (Souza and Fernandes, 2014).

The act of breastfeeding is multifactorial, that is, insufficient knowledge, culture and lack of self-efficacy can directly interfere with the early discontinuation of this practice (Dodt et al., 2012). Self-efficacy is the personal confidence that one can successfully accomplish what one wants, and the strategies used must be based on the four sources of information: personal experience, vicarious experience, verbal persuasion, physical and emotional state (Bandura et al., 1997). According to Oria and Ximenes (2010), it is necessary for the woman to have elements that can positively influence her choice to breastfeed, among which is the woman's perception of her confidence to breastfeed, which can be represented by the knowledge and skills that she judges enough to successfully breastfeed her baby. Therefore, it is believed that health professionals interventions can minimize and/or reduce the early weaning rates.

The aim of this study was to evaluate the perception of breastfeeding self-efficacy in puerperal women and to verify the association between Breastfeeding Self-Efficacy Scale-Brazilian Version (BSES-VB) scores with sociodemographic and obstetric variables.

## 2 Methods

This is a quantitative cross-sectional study that was developed in a Multiple Occupancy Rooming Unit of a public maternity hospital in the municipality of Santo André, São Paulo, Brazil, between June and September of 2016. The sampling was by convenience and non-probabilistic, constituted by 100 women of all age groups, who were in the puerperal period and attended that location.

The adopted inclusion criteria were puerperal period (12–72 hours postpartum), inpatients in the shared rooming unit and accompanied by the newborn (NB) in good health. Exclusion criteria were puerperal women with some cognitive and mental impairment, those with maternal infectious conditions that prevented or contraindicated breastfeeding, drug users and who were inpatients in the shared rooming unit, but with babies hospitalized in the Neonatal Intensive Care Unit. This study was approved by Institutional Ethics Committee (Protocol No. 1.614.201) and all participants signed Free and Informed Consent Form. Adolescents were all accompanied by a responsible one, and all of them signed the FICF.

In order to collect data, two steps were applied, a form that included sociodemographic, economic, obstetric, and breastfeeding variables through interviews. After this step, the Breastfeeding Self-Efficacy Scale-Brazilian Version (BSES-VB) was delivered to the puerperal mother to evaluate breastfeeding self-efficacy, in which the puerperal mother performed self-assessment. The original Breastfeeding Self-Efficacy Scale was written in English and it was translated to Portuguese for the convenience of women included in this study.

The *Breastfeeding Self-Efficacy Scale*-Brazilian Version (BSES-VB) is a Likert type scale, containing 33 questions, consisting of three dimensions (magnitude, generalization and strength) and is based on four sources of information (personal experience, observational or vicarious experience, verbal persuasion, and emotional and physiological state). Scale scores range from 33 to 165 points (Oria and Ximenes, 2010). Breastfeeding confidence is divided into low confidence or efficacy (33–118 points), medium confidence or efficacy (119–137 points), high confidence or efficacy (138–165 points) (Blyth et al., 2004).

A spreadsheet database was prepared in Excel 2016 for a descriptive and inferential analysis procedure and the information collected was exported and organized in the Epi Info version 7.0 program. To verify the relationship between BSES-VB with age, socioeconomic conditions, gestational and current history, the Kruskal Wallis test was performed. To verify the association between variables and items of the breastfeeding self-sufficiency scale (BSES-VB), the Mantel Haenszel and chi-square test were performed. Statistical significance was set at 5% for all analyzes.

## 3 Results

The sample was established with 100 puerperal women in the breastfeeding phase. The socio-demographic characteristics of these participants can be found in Table 1. Obstetric variables were not associated with maternal breastfeeding self-efficacy. Distribution of health variables, gestational history and current gestation of puerperal women included in this study can be found in Table 2.

**Table 1:** Characteristics of puerperal women included in this study.

Variables		n^a^	%	Mean	±	SD
Age	15–19	18	18.0	26,1	15/43	6.645
	20–34	71	71.0			
	35–43	11	11.0			
People living in the residence	2 to 5	74	74.0	4.5	02/10	1.636
	6 to 10	26	26.0			
Marital status	Married/civil partnership	63	63.0	–	–	–
	Single	36	36.0			
	Divorced	1	1.0			
Education	Tertiary	16	16.0	–	–	–
	SS C/I	61	61.0			
	Complete ES	18	18.0			
	Incomplete ES	5	5.0			
Occupation	Housewife	46	46.0	–	–	–
	Student	6	6.0			
	Employed	31	31.0			
	Self-employed	17	17.0			
Family income	>5 MW	1	1.0	–	–	–
	4 to 5 MW	20	20.0			
	2 to 3 MW	60	60.0			
	1 to <1 MW	19	19.0			

a Missing data is excluded; MW- minimum wage (approx. 250US$/month); C/I- complete/incomplete; SS- secondary school; ES- elementary school; SD- standard deviation.

**Table 2:** Distribution of health variables, gestational history and current gestation of puerperal women at a Public Municipal Hospital.

Variables		n	%	Mean	±	SD
Smoker	Yes	4	4	–	–	–
	No	85	85,0			
	Ex-smoker	11	11,0			
Cigarettes/day	2–7	6	6,0	11.0	2.0/20.0	7.043
	8	89	89,0			
	15–20	5	5,0			
Alcohol consumer	No	81	81,0	–	–	–
	Yes	13	13,0			
	No longer drinking	6	6,0			
Drug user	No	94	94,0	–	–	–
	Yes	6	6,0			
No. pregnancies	Primigravida	33	33,0	2.3	1.0/10.0	1.523
	2–5	64	64,0			
	6–10	3	3,0			
Parity	Primipara	40	40,0	2.1	1.0/8.0	1.343
	2–5 births	57	57,0			
	6–8 births	3	3,0			
Live births	1–3	85	85,0	2.1	1.0/8.0	1.353
	4–8	15	15,0			
Miscarriages	Yes	17	17,0	–	–	–
	No	83	83,0			
Prenatal consultations	>6	68	68,0	–	–	–
	6	19	19,0			
	1–5	13	13,0			
Received guidance on Breastfeeding	Yes	55	55,0	–	–	–
	No	45	45,0			
Type of delivery	Vaginal	62	62,0	–	–	–
	C-section	38	380			

Among all women included in this study, 67% were multigravida and 33% were primigravida. Table 3 shows that most of multigravida puerperal women reported having exclusively breastfeeding their previous children (81.7%). Among the main causes for exclusively breastfeeding were prevention of diseases/health (25%), love/pleasure (14%). The main cause for not exclusively breastfeeding was difficulty in the management of breastfeeding (11%). When questioned about the intention to breastfeed, 98% answered yes and 2% answered no. Of the women who intended to breastfeed, 68% intend to do so for more than six months, 28% from four to six months and 8% from two to three months.

**Table 3:** Distribution of variables on the practice of prior breastfeeding practice of puerperal women at a Public Municipal Hospital. Santo André, 2016.

Variables		n	%
Exclusively breastfed^a^	Yes	49	81.7
	No	11	18.3
Exclusive breastfeeding duration^a^	5–6 months	28	57.1
	3–4 months	13	26.5
	1–2 months	2	4.1
	Less than 1 month	6	1.2
Reasons to exclusively breastfeed^b^	Love/pleasure	14	14.0
	Prevention of diseases/health	25	25.0
	Financial/safety	8	8.0
	Benefits of breast milk/natural	6	6.0
Reasons not to exclusively breastfeed^a^	Baby's development/other	3	3.0
	Difficulty in the management of breastfeeding	11	11.0
	NB was in the neo ICU	2	2.0
	Lack of time/work	4	4.0
Intention to breastfeed	Yes	98	98.0
	No	2	2.0
Intended breastfeeding duration	>6 months	62	62.0
	4–6 months	28	28.0
	2–3 months	8	8.0
	Does not want to breastfeed	2	2.0
Intends to return to work	Yes	69	69.0
	No	31	31.0
Intended time to return to work	Works at home	4	4.0
	NB with more than 6 months	30	30.0
	NB with 5–6 months	25	25.0
	NB with 1–4 months	10	10.0

a Excluding primigravidas.

b More than one answer per subject NB = newborn.

In the sample studied, 40% of mothers had scores compatible with medium self-efficacy for breastfeeding (119–137 points), 35% of the puerperals presented high efficacy and 25%, low efficacy.

Table 4 presents the responses corresponding to the domains regarding breastfeeding technique and interpersonal aspects. Regarding the technical domain, the greatest difficulty presented is the perception about the amount of milk that the NB is receiving (28%), followed by a low degree of confidence in the adequate production of breast milk (24%). It is also worth noting that 20% of puerperal women lacked confidence to breastfeed in public places. Regarding the interpersonal domain, non-confidence in performing other tasks during the breastfeeding process was reported by 28%, followed by uncertainty about breastfeeding satisfaction (20%). Still, related to the interpersonal domain, 19% of the puerperal mothers find it difficult to believe that breastfeeding can occur for a month and a half.

**Table 4:** Responses of puerperal mothers of a Municipal Public Hospital during the application of the BSES-VB Scale by domains.

Breastfeeding Self-Efficacy Scale (BVSE-VB)^a^	Not confident at all n	Not very confident n
Technical domain		
1. I can always hold my baby comfortably during breastfeeding	1	3
2. I can always position my baby correctly at my breast	1	3
4. I can always recognize the signs of a latch	0	6
5. I can always take my baby off the breast without pain to myself	1	15
6. I can always determine that my baby is getting enough breast milk	4	20
10. I can always monitor how much breast milk my baby is getting by keeping track my baby's urine and bowel movements	6	22
12. Can I always ensure that my baby is properlylatched for the whole feeding	1	4
14. I can always manage to breastfeed even if my baby is crying	1	14
15. I can always keep my baby awake at my breast during feeding	2	15
16. I can always maintain my milk supply by using the “supply and demand” rule	9	14
18. I can always feed my baby with breastmilk only	3	5
22. I can always feed my baby every 2–3 hours	1	2
26. I can always comfortably breastfeed in public spaces	6	14
28. I can always finish breastfeeding my baby on one breast before switching to the other breast	1	4
29. I can always continue to breastfeed my baby for every feeding	1	5
30. I can always feel if my baby is sucking properly at my breast	2	7
31. I can always accept the fact that breastfeeding temporarily limits my freedom	11	6
32. I can always manage to keep up with my baby breastfeeding demands	3	2
33. I can always tell when my baby is finished breastfeeding	3	11
Intrapersonal domain		
3. I can always focus on getting through one feed at a time	3	4
7. I can always successfully cope with breastfeeding like I have with other challenging tasks	9	13
8. I can always depend on my family to support my decision to breastfeed	5	3
9. I can always motivate myself to breastfeeding successfully	3	9
13. I can always manage the breastfeeding situation to my satisfaction	5	15
17. I can always refrain from bottle feeding for the first 4 weeks	6	8
19. I can always stay motivated to breastfeeding my baby	1	3
20. I can always count on my friends to support my breastfeeding	1	3
21. I can always keep wanting to breastfeed	1	5
23. I can always keep feeling that I really want to breastfeed my baby for at least 6 weeks	9	10
24. I can always comfortably breastfeed with my family members present	5	12
25. I can always be satisfied with my breastfeedind experience	1	8
27. I can always deal with the fact that breastfeeding is time consuming	3	7

a Only answers that mean lower breastfeeding confidence.

Table 5 shows the relationship between some items of BVES-VB scale and variables of the study, shown by the chi-square test. This table displays the tests that show statistical significance. Namely, in the variable “breastfeeding guidance in prenatal care”, mothers proportionately show higher self-efficacy or efficacy with items 6, 16 and 26. Another variable observed was the “marital status”, proportionally, mothers who have a partner have higher breastfeeding self-efficacy.

**Table 5:** Association with maternal variables and questions of the breastfeeding self-efficacy scale Brazilian version (BSES-VB) applied in women who had given birth at a Public Municipal Hospital. Santo André, 2016.

Variables		Scale responses 3,4,5^a^	Scale responses 1 e 2^a^	x^2^	p Value^c^
		n^b^ (%)	n^b^ (%)		
**Technical domain**					
**Question 6**					
Prenatal breastfeeding guidance	Yes	46 (83.6)	9 (16.4)	4.141	0.042
	No	29 (65.9)	15 (34.1)		
**Question 15**					
Marital status	Has a partner	56 (9.3)	6 (9.7)	6.485	0.011
	Does not have a partner	26 (70.3)	11 (29.7)		
**Question 16**					
Prenatal breastfeeding guidance	Yes	49 (89.1)	6 (10.9)	9.071	0.003
	No	28 (63.6)	16 (36.4)		
**Question 26**					
Prenatal breastfeeding guidance	Yes	49 (89.1)	6 (10.9)	5.419	0.021
	No	31 (70.5)	13 (29.5)		
**Intrapersonal domain**					
**Question 7**					
Marital status	Has a partner	54 (87.1)	8 (12.9)	6.485	0.011
	Does not have a partner	23 (62.2)	14 (37.8)		

Questions 6, 15, 16, 26, 7: I can always determine that my baby is getting enough breast milk; I can always keep my baby awake at my breast during a feeding; I can always maintain my milk supply by using the “supply and demand” rule; I can always comfortably breastfeed in public places; I can always successfully cope with breastfeeding like I have with other challenging tasks.

a Likert scale (1- not confident at all; 2-not very confident; 3-sometimes confident; 4-confident; 5-very confident).

b An outlier was excluded.

c Mantel Haenszel test. P ≤ 0.05.

## 4 Discussion

In this study, most of the puerperal mothers were between 20 and 34 years of age, had a good level of education, did not work outside the home, had a partner and had a low family income, with an average of 4.5 people living in the household. Some factors positively influence breastfeeding, among them: having a partner, family support and a higher level of schooling (Pradhan et al., 2015).

Monteiro et al. (2017) and Oliveira et al. (2013) suggest that the higher the schooling, the greater the prevalence of exclusive maternal breastfeeding, thus, women with less schooling have less access to health services and family support, factors that may contribute to early weaning.

According to the obstetric variables, 57% were multiparous, but 40% were primigravidae, and 83% had no history of miscarriages, in the present study. According to Bandura et al. (1997), previous experiences are a source for increased self-efficacy. Thus, obstetric history should be considered relevant to cope with a new pregnancy (Dodt et al., 2013).

The number of prenatal consultations in 87% of the women was six and more consultations; however a large number of them did not receive guidance regarding breastfeeding in prenatal care. The Ministry of Health recommends that guidance, encouragement and support for breastfeeding should be carried out mainly during prenatal care to avoid excessive information during the puerperal period and for the mother to be more secure at this time of great transformation.

It is also observed that most of deliveries were vaginal (62%) versus 38% of cesarean section, even so, still far higher than the 15% recommended by WHO (MS-BR, 2006).

Regarding the exclusive breastfeeding of the previous child, 81.7% of the women who exclusively breastfed, 6% breastfed for less than one month, 26.5% for three to four months and 57.1% for five to six months. In qualitative terms, the most prominent answer to the reason for breastfeeding was health and the prevention of diseases. Among the reasons for not previously breastfeeding was the difficulty managing breastfeeding, with 11%. Multiparous women that had previous positive experiences with breastfeeding had higher self-efficacy scores, but it cannot be said that this experience will maintain exclusive breastfeeding for the recommended time, since they may be influenced by myths and beliefs during the puerperal period or by a previous failure (Oria and Ximenes, 2010). Exclusive breastfeeding has been consistently recommended by health authorities such as the World Health Organization (WHO) during the first six months of the child's life and mixed feeding should be provided up to 24 months (WHO, 2003, 2006).

It should be noted that the BSES-VB consists of 33 items, with five response options (1–5 points), and the total scale score can vary between 33 (minimum value), 99 (medium value) and 165 (maximum value). Thus, it was verified that the puerperal mothers presented the following values: minimum of 71, average of 130 and maximum of 165. Scale application can predict whether the mother decides to breastfeed or not, how much effort she will put into breastfeeding, whether she will have self-reinforcing or self-sabotaging thoughts and how she can react emotionally to the difficulties to practice breastfeeding. Besides these, it is of great importance to know the causes of the early weaning and to be able to intervene and help increase the rates of breastfeeding (Dennis and Faux, 1999).

In the association with some variables and items of the breastfeeding self-efficacy scale, the variables referring to the breastfeeding guidance in the prenatal period and marital status were statistically significant with the mean scores of each item on the scale. Breastfeeding guidance with items 6, 16 and 26 with the values (p = 0.042), (p = 0.03) and (p = 0.021) respectively. Marital status with items 7 and 15, both presenting values (p = 0.011). The information provided to pregnant women during prenatal care is extremely important for the continuation of breastfeeding, especially in the first month, when several complications with milk letdown may occur and may become an aggravating factor for early weaning (Almeida et al., 2015). A randomized study conducted in Singapore revealed that prenatal education on the benefits and management of breastfeeding increases the prevalence of exclusive breastfeeding (Su et al., 2007). An important randomized clinical trial showed that the groups that received health education interventions on breastfeeding in prenatal exclusively breastfed for longer periods compared to the control groups (Mattar et al., 2007; Sandy et al., 2009).

Regarding marital status, Marques (2010) stated that the approval of breastfeeding by the father of the child was associated with the incidence of lactation, longer duration of breastfeeding and exclusive maternal breastfeeding, data that corroborate the positive influence of the father in this process. The scientific evidence on the subject clearly indicates that high levels of schooling and having a partner are significant variables for the practice of exclusive maternal breastfeeding (Ku and Chow, 2010).

The fact that the sampling in this study is convenience and non-probability may represent a limiting factor in the research. However, a non-probability sample is considered to be important to know the reality of the population that is being studied. In this sense, the relevant highlights of this study show that, from the initial moments of breastfeeding, in the immediate puerperal period, there are multifactorial influences, urging that further studies be carried out.

## 5 Conclusion

In the present study it was shown that only 35% of the puerperal mothers presented high breastfeeding efficacy. This result suggests that public educational policies should be more focused on this population. Furthermore, the results of the present study recommend the use of the BSES-VB instrument to identify puerperal women at risk of early weaning.

## Declarations

### Author contribution statement

Luciane Morelis de Abreu: Conceived and designed the experiments; Performed the experiments; Wrote the paper.

Rosângela Filipini: Conceived and designed the experiments; Contributed reagents, materials, analysis tools or data; Wrote the paper.

Beatriz da Costa Aguiar Alves, Gláucia Luciano da Veiga: Analyzed and interpreted the data; Wrote the paper.

Fernando Luiz Affonso Fonseca: Contributed reagents, materials, analysis tools or data; Wrote the paper.

### Funding statement

This research did not receive any specific grant from funding agencies in the public, commercial, or not-for-profit sectors.

### Competing interest statement

The authors declare no conflict of interest.

### Additional information

No additional information is available for this paper.
